# Ocular Biometry and Refractive Prediction in Short Eyes: A Comparison of Two Swept-Source Optical Coherence Tomography-Based Biometers

**DOI:** 10.3390/bioengineering12090983

**Published:** 2025-09-16

**Authors:** Jiyun Seong, Sang Beom Han

**Affiliations:** Saevit Eye Hospital, 1065 Jungang-ro, Ilsandong-gu, Goyang 10447, Republic of Korea

**Keywords:** biometry, cataract, refractive error, SS-OCT

## Abstract

***Purpose:*** To compare the performance of two swept-source optical coherence tomography-based biometers in the measurement of ocular biometry and the prediction of postoperative refractive errors in eyes with short axial length (AL). ***Methods:*** A total of 48 eyes from 29 patients with AL ≤ 22 mm were included. AL, anterior chamber depth (ACD), keratometry (K), and lens thickness (LT) measured using the IOLMaster^®^ 700 and ARGOS^®^ before cataract surgery were compared. The refractive error prediction accuracy of the two devices was also compared. ***Results:*** This study included four men (7 eyes) and 25 women (41 eyes), with an average age of 70.7 ± 8.1 years (mean ± SD; range, 47–82 years). The two devices demonstrated good agreement in measurements of ocular biometry with high intraclass correlation coefficients (AL = 0.975; ACD = 0.957; K = 0.988; LT = 0.994). However, AL and ACD were significantly shorter when measured with the IOLMaster^®^ 700 compared to the ARGOS^®^ (*p* < 0.001 for both). There was no significant difference in mean absolute prediction errors between the two devices (*p* = 0.423). The IOLMaster^®^ 700 showed a significantly lower mean prediction error than the ARGOS^®^ (+0.12 ± 0.39 diopters vs. +0.20 ± 0.39 diopters, *p* = 0.006), although the difference was of limited clinical relevance. There were no significant differences in the percentages of eyes within ± 0.50 D (77.1% vs. 75.0%, *p* = 0.811) and ± 1.00 D (100% vs. 97.9%, *p* = 0.315) of the predicted refractive error. ***Conclusions:*** Although IOLMaster^®^ 700 and ARGOS^®^ showed good agreements in eyes with short AL, significant differences were observed in the measurements of AL and ACD. Both devices demonstrated good efficacy and comparable performance in predicting postoperative refractive errors.

## 1. Introduction

Cataract surgery has evolved beyond simple extraction of the opacified lens to become a refractive procedure aimed at enhancing visual performance and reducing dependence on corrective eyewear [1,2,3]. To achieve optimal visual outcomes and spectacle independence, precise prediction of postoperative refractive error through accurate determination of IOL power is necessary [3].

Accurate IOL power calculation requires precise measurement of ocular biometric parameters, including axial length (AL), anterior chamber depth (ACD), keratometry (K), and lens thickness (LT) [4,5]. In short eyes, minor measurement inaccuracies can result in disproportionately large postoperative refractive errors [6]. In addition, the characteristics of short eyes, such as a shallow AC, relatively greater LT, and a steep cornea, can often result in inaccurate prediction of effective lens position (ELP) [6,7], which may lead to increased postoperative refractive errors. Therefore, precise measurement of these parameters is essential for achieving optimal refractive outcomes [3,6].

Until recently, devices based on partial coherence interferometry have been widely used for ocular biometry measurements and IOL power calculations [3]. However, these devices are limited in their ability to measure AL in eyes with severe media opacities, such as dense posterior or anterior subcapsular cataracts or mature cataracts [3]. More recently, swept-source optical coherence tomography (SS-OCT)-based devices have been rapidly adopted in clinical practice [3]. These devices, such as the IOLMaster^®^ 700 (Carl Zeiss Meditec, Jena, Germany) and ARGOS^®^ (Movu, Komaki, Japan), employ a light source with a narrow bandwidth, resulting in improved tissue penetration and reduced scattering effects caused by media opacities [8,9,10]. By enhancing image quality, these devices provide more accurate visualization of posterior structures, including the fovea [8,9,11,12]. This enables precise measurement of distances between intraocular structures and better monitoring of patient fixation, leading to improved success rates and accuracy of ocular biometry, even in cases of dense cataracts and poor fixation [3,8,9,11,12,13]. Consequently, the application of SS-OCT-based ocular biometry devices offers potential advantages for improving visual and refractive outcomes in eyes with short AL [14,15].

Although the IOLMaster^®^ 700 and ARGOS^®^ acquire ocular biometry in a similar manner, there is a fundamental difference between the two devices: the IOLMaster^®^ 700 uses a single, equivalent refractive index across the entire eye, whereas the ARGOS^®^ applies different refractive indices for each segment, such as AL, K, ACD, and LT [11,12,16].

Several studies have compared the biometric measurements and refractive prediction errors between IOLMaster^®^ 700 and ARGOS^®^ [3,10,17,18,19,20]. However, to the best of our knowledge, only two studies have included the data from eyes with short AL [3,17]. Both studies analyzed data from eyes < 22.5 mm, potentially incorporating borderline cases with ALs ranging from 22.0 to 22.5 mm [3,17]. A discrepancy exists between the results of the two studies. For instance, Porwolik et al. [17] reported that IOLMaster^®^ 700 measured a significantly shorter ACD compared to ARGOS^®^, whereas Yang et al. [3] found no significant difference in ACD measurements between the two devices. Moreover, Porwolik et al. [17] did not evaluate refractive predictive accuracy.

Therefore, we conducted this study to compare ocular biometric measurements obtained using the IOLMaster^®^ 700 and ARGOS^®^ in eyes with short AL (defined as AL ≤ 22 mm). In addition, we compared the predictive accuracy of postoperative refractive outcomes calculated by each device.

## 2. Methods

### 2.1. Ethics Statement

This study was approved by the Institutional Review Board (IRB) of Saevit Eye Hospital (IRB No. B SVEC 202504-001-01) and adhered to the tenets of the Declaration of Helsinki. The waiver of informed consent was approved by the IRB because this study did not include any identifiable patient information.

### 2.2. Study Participants

In this retrospective study, medical records of patients who received preoperative evaluation for cataract surgery between October 2024 and April 2025 at Saevit Eye Hospital were reviewed. For each patient, ocular biometric parameters, such as AL, ACD, LT and K, were measured using both the IOLMaster^®^ 700 and ARGOS^®^ on the same day. Only patients with AL measurements of ≤22 mm on both devices were consecutively included in this study.

The exclusion criteria included corneal diseases that could affect corneal biometry (e.g., pterygium, corneal opacity, and corneal nodules), severe lens opacity that rendered measurements with the IOLMaster^®^ 700 or ARGOS^®^ impossible, retinal pathologies that might interfere with biometric measurements (e.g., epiretinal membrane and macular edema), history of ocular surgery or trauma, and intraoperative complications including capsular tear or rupture and zonular dialysis. For each patient, the IOL power was determined with the target refraction of 0 ± 0.5 diopters using Barrett Universal II (BUII) formula, which has shown relatively high accuracy in eyes with short AL [21,22]. The SN60AT IOLs (Alcon Laboratories, Inc., Fort Worth, TX, USA) were implanted in all the participants and therefore were used as the model for IOL calculations.

Demographic data including age and sex and ocular biometric parameters including AL, ACD, K, and LT measured by each device were collected and analyzed. Refractive prediction errors were evaluated based on manifest refraction results at 1 month postoperatively. For each patient, the mean arithmetic prediction error (ME) was calculated by subtracting the target refraction predicted by each device from the spherical equivalent at postoperative 1 month [1,2]. The ME, mean absolute prediction error (MAE), and the percentages of eyes with arithmetic prediction errors within ± 0.50 diopter (D) and ± 1.00 D were calculated and analyzed.

### 2.3. Statistical Analysis

Statistical analyses were performed using SPSS software version 22.0 (SPSS, Inc., Chicago, IL, USA). All continuous variables were expressed as mean ± standard deviation (SD). Agreement between the two devices was assessed using the intraclass correlation coefficient (ICC). Bland-Altman plots were generated to assess the agreement between the devices. Paired t-tests were used to assess differences in each biometric parameter and refractive prediction errors between the two devices [16]. Percentage of eyes with a prediction error within ± 0.50 D and ± 1.00 D were analyzed with Chi-square test. *p* values < 0.05 were considered statistically significant.

## 3. Results

This study included 48 eyes of 29 patients, comprising 4 men (7 eyes) and 25 women (41 eyes) (Table 1). The average age was 70.7 ± 8.1 years (mean ± SD; range, 47–82 years).

**Figure 1:**
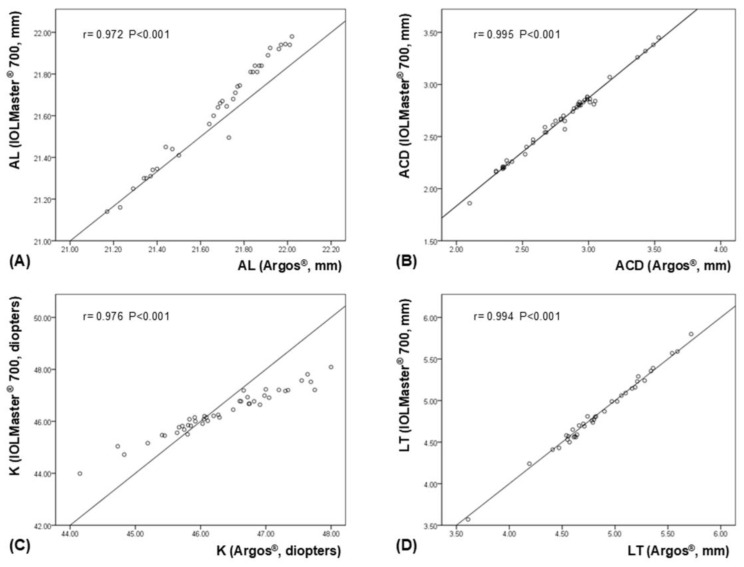
Scatterplots depicting significant correlations between the ARGOS^®^ and IOLMaster^®^ 700 for each ocular biometric parameter. (**A**) axial length (AL), (**B**) anterior chamber depth (ACD), (**C**) keratometry (K), and (**D**) lens thickness (LT).

**Figure 2:**
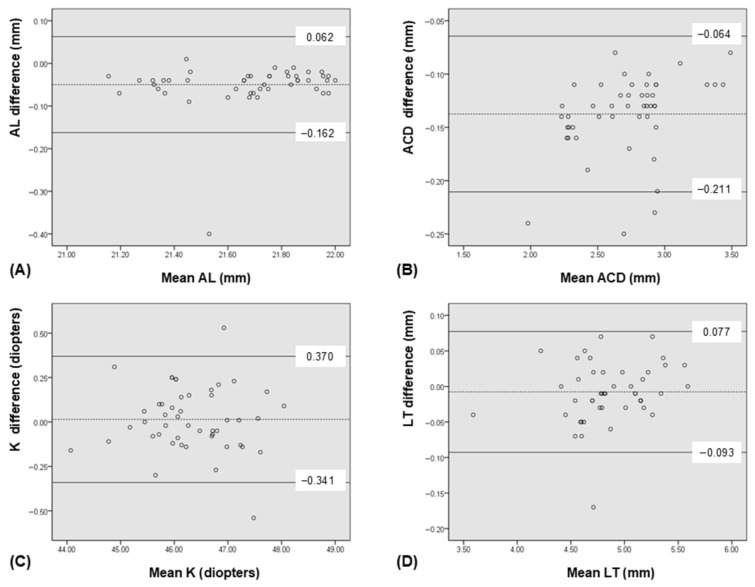
Bland-Altman plots demonstrating the good agreement between the ARGOS^®^ and IOLMaster^®^ 700 foreach ocular biometric parameter. The mean difference is indicated by the dashed lines, and 95% limit of agreement (LoA) is indicated by the solid line. (**A**) axial length (AL), (**B**) anterior chamber depth (ACD), (**C**) keratometry (K), and (**D**) lens thickness (LT).

The ICCs for the biometric measurements obtained using the IOLMaster^®^ 700 and ARGOS^®^ were 0.975 for AL, 0.957 for ACD, 0.988 for K, and 0.994 for LT, indicating a high level of agreement between the two devices across all parameters. Regarding the comparison of each parameter, ARGOS^®^ showed significantly longer AL measurements than did the IOLMaster^®^ 700 (*p* < 0.001). ACD measured with the ARGOS^®^ was also significantly greater than that measured with the IOLMaster^®^ 700 (*p* < 0.001). There were no significant differences in K (*p* = 0.585) and LT (*p* = 0.117) measurements between the two devices (Table 2). Scatterplots demonstrated significant correlations for AL, ACD, K and LT between the two devices (Figure 1). Bland–Altman plots showed good agreement for AL, ACD, LT and K between the devices (Figure 2).

Regarding refractive error prediction, the IOLMaster^®^ 700 demonstrated a significantly lower ME compared to the ARGOS^®^ (0.12 ± 0.39 D vs. 0.20 ± 0.38 D, *p* = 0.006). There was no significant difference in MAE (0.32 ± 0.25 D vs. 0.34 ± 0.25 D, *p* = 0.423) between the two devices.

There were no significant differences between the IOLMaster^®^ 700 and ARGOS^®^ in the percentages of eyes within ± 0.50 D (77.1% vs. 75.0%, *p* = 0.811) and ± 1.00 D (100% vs. 97.9%, *p* = 0.315) of target refraction, respectively (Table 3).

**Figure 3:**
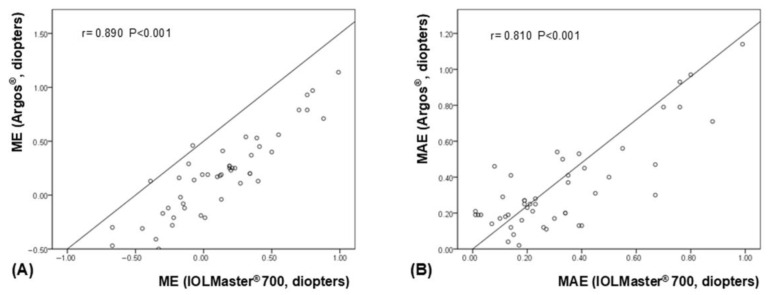
Scatterplots illustrating the significant correlations between refractive prediction errors obtained using the ARGOS^®^ and IOLMaster^®^ 700. (**A**) mean arithmetic prediction error (ME), (**B**) mean absolute prediction error (MAE).

**Figure 4:**
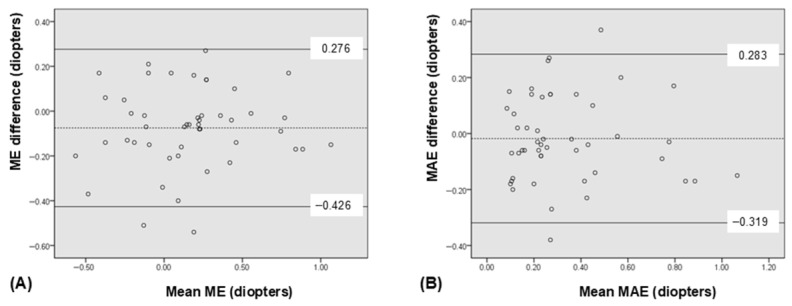
Bland-Altman plots depicting good agreement between the ARGOS^®^ and IOLMaster^®^ 700 in refractive prediction errors. The mean difference is indicated by the dashed lines, and 95% limit of agreement (LoA) is indicated by the solid line. (**A**) mean arithmetic prediction error (ME), (**B**) mean absolute prediction error (MAE).

Both ME and MAE measured using IOLMaster^®^ 700 and ARGOS^®^ showed significant correlations (*p* < 0.001 for both, and ICC = 0.874 and 0.811, respectively). Scatterplots showed significant correlations in both ME and MAE (Figure 3). The relevant Bland-Altman plot is shown in Figure 4.

## 4. Discussion

The results of the present study indicate that ocular biometric parameters measured using the two SS-OCT-based devices exhibit a high degree of correlation in short eyes with an AL ≤ 22 mm. However, ACD and AL were measured significantly shorter with the IOLMaster^®^ 700 than with the ARGOS^®^, suggesting that these two devices may not be interchangeable for biometric measurements in short eyes.

Previous studies also reported that ACD was significantly greater when measured with the ARGOS^®^ than with the IOLMaster^®^ 700 [10,17,18], although Yang et al. [3] found no significant difference in ACD measurements between the two devices. Porwolik et al. [17] reported that AL measured with the ARGOS^®^ was significantly longer compared to the IOLMaster^®^ 700, which is consistent with the results of this study. Similarly, Yang et al. [3] found that AL measured with the ARGOS^®^ was significantly longer in short eyes with AL < 22.5 mm and was significantly shorter in long eyes with AL > 26.0 mm when compared to measurements obtained with the IOLMaster^®^ 700. Other studies have reported similar findings, demonstrating that AL is measured significantly shorter in short eyes and longer in long eyes with the IOLMaster^®^ 700 compared to the ARGOS^®^ [15,19].

Although both devices measure ocular biometry based on the SS-OCT principle, the IOLMaster^®^ 700 uses a uniform refractive index of 1.3375 across the entire eye, whereas the ARGOS^®^ applies different refractive indices for each ocular component: 1.376 for the cornea, 1.336 for the aqueous humor, 1.410 for the lens, and 1.336 for the vitreous body. This difference in refractive index application may contribute to discrepancies in measurements of ocular biometry between the two devices [3,10]. In average eyes, the results of AL measurement may not be different between the two devices because of calibration integrated into the devices [7,17]. However, in short eyes, the LT is relatively greater in proportion to the AL and anterior segment, and this disproportion becomes more pronounced as AL decreases [7]. Consequently, discrepancies may occur between the devices that assume a single refractive index throughout the whole eye and those that apply segment-specific refractive indices for different ocular structures [7,10,17,23].

Accurate measurement of AL is particularly critical, as a 1 mm error in the AL can result in a 2.5 to 3.0 D error in the calculated IOL power [5,24]. Errors in the ACD measurement may also cause inaccuracies in IOL power calculation due to incorrect prediction of ELP [6,7], potentially leading to increased postoperative refractive errors in short eyes [25]. However, despite the differences in ACD and AL measurements between the IOLMaster^®^ 700 and ARGOS^®^, no significant differences were observed in MAE between the two devices. These findings suggest that the internal calibration mechanisms of both devices might contribute to minimizing prediction errors. However, ME was statistically significantly lower when measured with the IOLMaster^®^ 700, although the difference (-0.08 D) does not appear to be clinically relevant. This suggests that the discrepancies in AL and ACD measurements might have contributed to the observed differences in ME, albeit small, warranting further investigation.

Yang et al. [3] reported no significant difference in refractive outcomes between the IOLMaster^®^ 700 and ARGOS^®^ in eyes with AL < 22.5 mm, which is not entirely consistent with the findings of the present study. Other studies have also reported comparable prediction accuracies between the IOLMaster^®^ 700 and ARGOS^®^ in normal or long eyes [3,20,26,27] Shammas et al. [19] reported that the MAEs obtained with ARGOS^®^ were significantly lower than those with the IOLMaster^®^ 700, which contradicts our findings. Similarly, Wang et al. [15] also demonstrated that ARGOS^®^ was associated with significantly lower MAEs and a greater percentage of eyes within ±0.50 D of error, which is also inconsistent with our findings. These discrepancies highlight the need for further studies to evaluate and compare the refractive predictive accuracy of these two devices.

In the present study, approximately 75% and 100% of eyes demonstrated refractive prediction errors within ± 0.50 D and ±1.00 D, respectively (Table 3), which are substantially higher than the percentages reported in previous studies [1,10,28]. Studies have consistently shown that eyes with short AL < 22 mm are associated with greater refractive prediction errors and a reduced likelihood of achieving postoperative refraction within ±0.50 D of the target [29]. Decreasing AL had a correlation with increasing postoperative refractive errors for all IOL formulae [29]. Although newer IOL formulae that incorporate ACD and LT might be helpful for improving the refractive outcome in short eyes, their prediction accuracy in short eyes is still inferior to that in eyes with normal AL [6]. More than 40% of eyes with AL < 21.5 mm exhibited a refractive prediction error greater than ±0.50 D, suggesting that achieving accurate refractive outcomes in short eyes remains a significant challenge despite advances in modern IOL calculation formulae [1,10]. Shivastava et al. [28] reported that the percentages of eyes with MAE within ±0.50 D and ±1.00 D ranged from 46% to 56% and from 76% to 80%, respectively, when ocular biometry was performed using a device based on partial coherence interferometry. The improved refractive prediction outcomes observed in the present study might be attributed to the enhanced accuracy and precision of SS-OCT-based devices compared to those used in previous studies [3,8,9,11−15]. A recent study reported that over 75% of eyes with a short AL < 22.5 mm demonstrated prediction errors within ± 0.5 D when IOL calculation was performed using ARGOS^®^ and BUII formula [14], supporting this assumption.

It is noteworthy that both ME and MAE obtained using the two devices demonstrated significant correlations. This finding may reflect the overall high accuracy of each device in predicting postoperative refractive outcomes, whereby cases with greater prediction errors in one device also tend to exhibit similar errors in the other.

This study has limitations as follows: (1) A relatively small number of eyes (48 eyes from 29 patients) were included in this retrospective study, as it was limited to short eyes with an axial length (AL) ≤ 22 mm. It is also remarkable that the male-to-female distribution (4:25) appears to be notably unbalanced, although patients have been consecutively and non-selectively recruited. Studies conducted in various countries have consistently shown that males have significantly longer axial lengths—approximately 0.5 mm—than females [30,31,32]. As a result, females are more likely to have a short axial length (≤22 mm), which may partly explain the observed sex ratio imbalance in the present study. This study also included patients with a relatively homogeneous ethnic and environmental background, although the contribution of ocular biometric parameters, such as AL, to refractive errors can be influenced by population-specific environmental factors [33]. Therefore, we believe that further prospective research with larger cohorts from diverse ethnic and environmental backgrounds is warranted to more comprehensively assess and validate the refractive prediction performance of the two devices. (2) Only a single formula, BUII, was used in the prediction of refractive errors. It is because we considered the BUII might be the most reliable formula for short eyes because it demonstrated the smallest prediction error compared to other formulae in eyes with short AL [21,34]. Ma et al. [35] reported that BUII outperformed other formulae, including Kane, Olsen, EVO, Hoffer Q, and Haigis, in Chinese eyes with AL ≤ 22.5 mm. Rementería-Capelo et al. [14] also found that ARGOS^®^ exhibited good refractive accuracy using the BUII in eyes with short AL < 22.5 mm [14]. However, we still believe the restriction to a single IOL formula substantially limits the generalizability of the results. Therefore, we believe further studies evaluating modern formulae, such as Kane, Hill-RBF, Olsen, or EVO, are necessary to optimize refractive outcomes, as there is a possibility that application of novel formulae could further improve refractive outcomes. (3) Refractive outcomes were reported only at 1 month, which might be insufficient to evaluate stable results. Further studies with a longer follow-up period of at least 3 to 6 months are therefore warranted.

In conclusion, although the IOLMaster^®^ 700 and ARGOS^®^ exhibited a high level of agreement in eyes with short AL ≤ 22 mm, ACD and AL measured with IOLMaster^®^ 700 were significantly shorter compared to those obtained using ARGOS^®^. Both SS-OCT-based devices demonstrated comparable performance and good accuracy in predicting postoperative refractive errors.

## Figures and Tables

**Table 1:** Age and sex distribution of the study participants.

	Male (*n*, (Eyes))	Female (*n*, (Eyes))
40–49 years		1 (1 eyes)
50–59 years		1 (1 eyes)
60–69 years	3 (5 eyes)	8 (13 eyes)
70–79 years	1 (2 eyes)	10 (18 eyes)
80 years or older		5 (8 eyes)
Total	4 (7 eyes)	25 (41 eyes)

**Table 2:** Comparison of ocular biometric parameters between IOLMaster^®^ 700 and ARGOS^®^.

Parameters	IOL Master^®^ 700	ARGOS	Mean Difference	95% CI *	*p* Value ^†^	ICC ^‡^
AL (mm)	21.65 ± 0.24	21.70 ± 0.23	−0.05	−0.07, −0.03	<0.001	0.975
ACD (mm)	2.65 ± 0.34	2.79 ± 0.33	−0.14	−0.15, −0.13	<0.001	0.957
K (diopters)	46.33 ± 0.81	46.32 ± 0.82	+0.01	−0.04, +0.07	0.585	0.988
LT (mm)	4.88 ± 0.39	4.89 ± 0.38	−0.01	−0.02, +0.00	0.224	0.994

Values are presented as mean ± SD. * 95% confidence interval of the mean difference (IOL Master^®^ 700—ARGOS^®^). ^†^ paired *t*-test. ^‡^ intracorrelation coefficient.

**Table 3:** Comparison of refractive prediction errors between IOL Master^®^ 700 and ARGOS^®^.

Parameters	IOL Master^®^ 700	ARGOS^®^	Mean Difference	95% CI *	*p* Value
ME ^†^ (diopters, D)	+0.12 ± 0.39	+0.20 ± 0.38	−0.08	−0.13, −0.02	0.006 ^§^
MAE ^‡^ (D)	0.32 ± 0.25	0.34 ± 0.25	−0.02	−0.06, +0.03	0.423 ^§^
Eyes within ± 0.50 D	77.1% (37/48)	75.0% (36/48)			0.811 ^∥^
Eyes within ± 1.00 D	100% (48/48)	97.9% (47/48)			0.315 ^∥^

Values are presented as mean ± SD. * 95% confidence interval of the mean difference (IOL Master^®^ 700—ARGOS^®^). ^†^ Mean error. ^‡^ Mean absolute error. ^§^ Paired *t*-test. ^∥^ Pearson’s Chi square test.

## Data Availability

The raw data supporting the conclusions of this article will be made available by the authors on request.
